# Association between using social media WeChat and depressive symptoms among middle-aged and older people: findings from a national survey

**DOI:** 10.1186/s12877-022-03054-y

**Published:** 2022-04-22

**Authors:** Xing Qu, Shannon H. Houser, Jian Zhang, Jin Wen, Wei Zhang

**Affiliations:** 1 Institute of Hospital Management, West China Hospital, Sichuan University, Chengdu, 610041 China; 2 Department of Health Services Administration, University of Alabama at Birmingham, Birmingham, AL USA; 3 West China Biomedical Big Data Center, West China Hospital, Sichuan University, Chengdu, 610041 China; 4 Medical Big Data Center, Sichuan University, Chengdu, 610041 China; 5 Mental Health Center, West China Hospital, Sichuan University, No. 37, Guoxue Lane, Chengdu, 610041 China

**Keywords:** Depressive symptoms, Mental health, Social media, WeChat, Elderly adults

## Abstract

**Objectives:**

We aimed to assess the characteristics and health status of a study sample using social media WeChat and to identify the association between social media usage and depressive symptoms among people aged 45 and older in China.

**Methods:**

Data were drawn from the China Health and Retirement Longitudinal Study (CHARLS). Depressive symptoms were measured by the 10-item form of the Center for Epidemiologic Studies Depression Scale (CES-D-10). The propensity score matching method (PSM) was performed to balance the characteristics of WeChat users and non-WeChat users. Multilevel logistic regression was used to test the association between the incidence of depressive symptoms and WeChat usage by introducing covariates step by step. Sensitivity analysis was conducted to estimate the robustness of the primary findings.

**Results:**

A total of 5415 matching cases out of 11,338 total sample were used in this study to generate the final analysis. A multilevel logistic regression model showed that a significantly lower incidence of depression was related to WeChat usage after adjusting for all possible covariates (OR: 0.76, 95% CI: 0.62–0.94). The most popular WeChat functions used by the study population were watching news (80.4%), posting Moment messages (75.5%), chatting with friends (66.0%), and watching videos (65.2%). The sensitivity analysis yielded similar findings to the primary analyses.

**Conclusions:**

Using social media WeChat showed an association with lower depressive symptoms among people aged ≥45 and older in our study sample. Further studies need to be explored on the promotion and education of social media WeChat usage, targeting the improvement of mental health-related issues through social network connections.

**Supplementary Information:**

The online version contains supplementary material available at 10.1186/s12877-022-03054-y.

## Introduction

Depression is a common mental health disorder worldwide that affects both physical and psychological health. More than 264 million people of all ages suffer from depression globally, and depression became the third leading cause of disability in 2017 [1]. Among the aging population, depression is a risk factor related to disability, increased mortality, and more comorbidities [2]. In China, the reported prevalence of depressive symptoms ranges from 6.9–37.9% among adults [3–6]. With a progressively aging population in China, prevention, early identification, and management of depression in middle-aged and older adults have become increasingly important.

Since the development of Internet technology, social media has become a new dimension impacting the population’s mental health [7, 8]. The influence of social media on mental health is a double-edged sword. Some of the noticeable advantages of using social media include increased interactions with others and enhanced social support [9], access to health information [10], health promotion [11, 12], and perceived emotional support [13]. In contrast, using social media was also associated with negative impacts, such as poor sleep quality [14, 15], increased depression [15–18], anxiety [19, 20], fear [21], experiencing fatigue [20], alcohol, tobacco, and other drug use [22], and felt more isolated [23]. Most of the current studies were completed with social media based on Facebook, Twitter, Instagram, and YouTube, which are popular in Western countries.

Unlike the popularity of social media in Western countries, WeChat is a predominant multipurpose social media platform widely used in China, with over one billion monthly active users [24]. First released by the Chinese multinational company Tencent Holding Limited in January 2011, WeChat is not only a social media platform but also a supporting platform for multiple purposes. The functions in WeChat include chatting by text, verbal, or video, social photo sharing tools, interaction functions, seeking and receiving information, financial aspects such as spending online or paying offline, and public platform functions such as health checks during the COVID-19 pandemic and health education programs [25]. An increasing number of studies have reported the benefits and limitations of WeChat usage associated with users’ health issues. For instance, WeChat use was associated with suppressing stress life events and better sleep quality among undergraduate students [26, 27], depressed mood [28], and postpartum depression among new mothers [29]. The use of WeChat has also been identified with probable anxiety and depression among children, adolescents, and special health populations with small samples or during a specific period [30]. However, there is rare information about the association between depressive symptoms and WeChat use among middle-aged and elderly populations. Understanding this association is essential for developing public health strategies to improve mental health for the overall aging population in China.

In this study, we aimed to 1) assess the characteristics and health status of the study sample using social media WeChat among people aged 45 and older and 2) identify the association between social media WeChat usage and depressive symptoms among these people.

## Methods

### Study design and study sample

We performed a cross-sectional, secondary analysis using the data obtained from Wave 4 in 2018 of the China Health and Retirement Longitudinal Study (CHARLS). CHARLS is a national longitudinal survey started in 2011 aimed at representing residents in mainland China aged 45 and older. The survey collects high-quality representative panel data for a wide range of topics, including demographic characteristics, socioeconomic status, family relations, health, and healthcare. Using a multistage probability sampling method, Chinese residents aged 45 and above were randomly sampled in 2011 with 10,000 households and 17,500 individuals at the baseline survey. The same individuals were followed up every two years. More detailed sampling procedures and study methodology for CHARLS have been discussed in previous studies [31].

The inclusion criteria for this study were individuals aged ≥45 years with complete outcome variables. For a more accurate estimation of the effect of WeChat usage on depression, data in Wave 3 in 2015 were used to exclude the individuals who already had depression. The exclusion criteria were individuals who already had depressive symptoms in Wave 3. We excluded the individuals with depressive symptoms in 2015 to make an approximate causal effect by setting WeChat usage time before or at least at the same time as the depressive symptom onset.

### Depressive symptoms

Depressive symptoms were set as the binary outcome variable in this study. Individuals were examined for depressive status by the form of the Center for Epidemiologic Studies Depression Scale (CES-D-10) at each wave. The CES-D is a widely used self-report measure of depression symptomatology, especially in middle- and low-income countries [32, 33]. A total of 10 items in the CES-D-10 assess the feelings of the participants during the last week, such as annoyance, hopefulness, fear, loneliness, unhappiness, attention deficit, and sleep disorder. Each item is rated against a Likert scale with scores ranging from 0 to 3: “rarely or none of the time” (< 1 day), “some or a little of the time” (1–2 days), “occasionally or a moderate amount of the time” (3–4 days), and “most or all of the time” (5–7 days). The final score is cumulatively calculated and ranges from 0 to 30. The cut-off scores for depressive symptoms were ≥ 10 for this 10-item version [34]. In this study, the respondents who scored greater than or equal to ten on the CES-D-10 were categorized as having depressive symptoms, and those who scored lower than ten were categorized as without depressive symptoms.

### Social media WeChat usage

The independent variable was social media usage. WeChat usage represented social media usage in this study. In Wave 4 of CHARLS in 2018, the participants were asked “Do you use WeChat?” with an answer of “yes” or “no”. The participants who answered “yes” to WeChat use were defined as the WeChat user.

If individuals answered “yes” to WeChat use, then asked “Do you post messages through WeChat Moments?” with an answer of “yes” or “no”. Moments is a function of WeChat used to share photos or comments within WeChat users. We divided WeChat users into three groups to see how social media usage exerts divergent effects on mental health outcome: (1) Those with an answer of “no” to both questions assigned in group 1; (2) those with an answer of “yes” to WeChat usage but “no” to the Moments usage assigned in group 2, and (3) those with an answer of “yes” to both questions assigned in group 3.

The purpose of using the internet for other functions was also investigated. The choices included chat, watch the news, watch the videos, playing games, financial management, and others. We assessed the function level and type of WeChat usage in regard to depressive symptoms among middle-aged and older people. One WeChat function equaled to score 1, the total scores ranged from 0 to 6, with higher scores indicating higher levels of WeChat usage. Then, we divided Chat into social function; watching the news, watching the video, and playing games as entertainment function; financial and the others as daily-life function. Third, we put each WeChat function as an independent type to see if WeChat usage function exerts divergent effects on mental health.

### Covariates

Based on literature review, we controlled the possible covariates which were related to depression and were available in the CHARLS, including demographic information [2, 35] (gender, age, education level, household income, live in rural or urban area, marriage status, ethnic), self-reported general health status (continuous variable, 1 is very good and 5 is very poor) [36], life satisfaction (cumulative 5-degree score of life satisfaction with life-as-whole, health, marriage, children, and air quality, higher score represented lower satisfaction) [37], comorbidity physical diseases [38] (cumulative score of 13 selected self-reported noninfectious diseases), cognition status (measure by the brief Community Screening Instrument for Dementia (CSI-D), CSI-D ≤ 4 defined as cognition impairment, completed only in population aged > 60 years, this variable was used in sensitivity analysis) [39], activities of daily living skill (measured by Katz Index of Independence in Activities of Daily Living, ADL; continuous variable, the higher score represented more independent the respondents were), smoke (non-smoke, smoke) and drinking status (drink, non-drink), sleep at night (hour, continuous variable) and nap at noon status (minutes, continuous variable) [40], physical activities (days of vigorous/moderate/mild activities in a week) [41], social activities (cumulative score of 10 kinds of social activities) [2].

### Statistical methods

Sample characteristics were compared between the participants who were WeChat users by using Student’s t tests for continuous data or chi-squared tests for categorical data. Given the observational nature of the data, treatment (WeChat usage) allocation was not randomly assigned in the study population. Therefore, we performed propensity score matching (PSM) to reduce the risk of bias due to confounders, and the causal effects of the various treatment regimens on the outcomes could be more precisely estimated [42]. We estimated the propensity score for a binary dependent variable indicating treatment status by selecting a quadratic function of covariates to include in the estimation function of the propensity score. The nearest neighbor PSM method was used to construct a 1:6 matching group. The covariates adjusted in PSM included age, gender province, education, living area, marital status, race, and income category.

Since the individuals were clustered in the province, multilevel logistic regression was used to test the effect of WeChat usage on depression in individuals after PSM [43]. The individual was considered the first level, and each province was considered the second level. We repeatedly performed multilevel logistic three times by introducing the covariates mentioned above step by step for double adjustment in case of remaining imbalanced after PSM [44]. Model 1 estimated the effect of WeChat usage on depressive symptoms without any covariates. Model 2 estimated the effect adjusted by age, gender, education level, living area, marital status, race, and income category. Model 3 (full model) estimated the effect adjusted by smoking, drinking, general health status, life satisfaction score, disabilities, comorbidity number, sleep at night and noon, physical activities, social activities, and ADL, plus model 2.

In the sensitivity analysis, we performed three methods to test the robustness of results. The first one was using a traditional logistic regression in the matching group to estimate the effect of WeChat usage on depression. Second, we performed multilevel logistic regression with the individual who completed the CSI-D test and had no cognitive impairment (CSI-D > 4). All the adjustments were the same as in model three. Third, we considered borderline cases by changing the cutoff score for the determinant of depression from 10 to 9 or 11 in the multilevel logistic regression in the full model.

Odds ratios (ORs) and 95% confidential intervals (95% CIs) were reported. A value of *P* < 0.05 was considered statistically significant. All the data were analyzed by using STATA 14.0 (TX, USA).

## Results

**Fig. 1:**
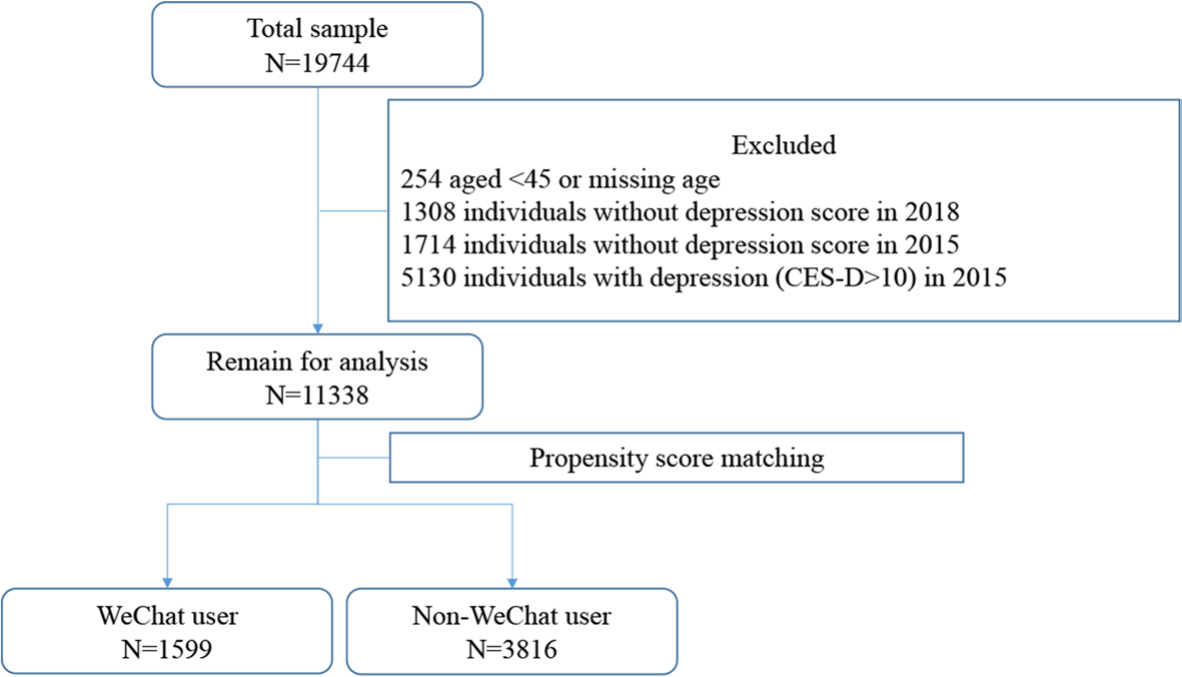
Data inclusion process

After data cleaning, 11,338 out of 19,744 cases qualified for analysis. Data inclusion process are shown in Fig. 1. After PSM, 1599 WeChat users and 3816 non-WeChat users were matched to generate the final analysis. Gender, race, and marital status were balanced between the WeChat user group and the non-WeChat user group. The difference in WeChat usage was persistently significant in age, urban or rural living area, education level, and income status after PSM. In the matching cases (*n* = 5415), mean age was 56.7 ± 7.7, 2376 (43.9%) were female, 3464 (64.0%) had middle or higher education level, 3250 (60.0%) lived in rural area, 3037 (56.1%) had equal or higher than medium income level. Among WeChat users, 211 (13.2%) had new onset depression symptoms, while 720 (18.9%) had new onset depression symptoms in the non-WeChat users. The other individual characteristics are documented in Table 1.

**Table 1:** Characteristics of sample before PSM and after PSM by WeChat users and non-WeChat users

	Before PSM (*N* = 11338)			After PSM (*N* = 5415)		
	WeChat user (*N* = 1632)	Non-WeChat user (*N* = 9706)	*P* value	WeChat user (*N* = 1599)	Non-WeChat user (*N* = 3816)	*P* value
With depressive symptoms (CES-D>10)	212 (13.0)	2140 (22.0)	< 0.001	211 (13.2)	720 (18.9)	< 0.001
Age	55.5 ± 7.3	62.8 ± 10.0	< 0.001	55.6 ± 7.3	57.2 ± 7.8	< 0.001
Female	697 (42.7)	4690 (48.3)	< 0.001	681 (42.6)	1695 (44.4)	0.22
Minority	111 (6.8)	741 (7.6)	0.23	110 (6.9)	254 (6.7)	0.77
Rural residence	764 (46.8)	7618 (78.5)	< 0.001	764 (47.8)	2486 (65.1)	< 0.001
Married	1541 (94.4)	8439 (86.9)	< 0.001	1511 (94.5)	3578 (93.8)	0.44
Middle school and higher education	1248 (76.5)	3143 (32.4)	< 0.001	1215 (76.0)	2249 (58.9)	< 0.001
Equal or higher than average income	1076 (65.9)	3376 (34.8)	< 0.001	1043 (65.2)	1994 (52.3)	< 0.001
Alcohol drinker	773 (47.4)	6368 (65.6)	< 0.001	837 (52.3)	1481 (38.8)	< 0.001
Smoker	1612 (98.8)	9565 (98.5)	0.11	1056 (66.0)	2535 (66.4)	0.89
Disabled	180 (11.0)	1583 (16.3)	< 0.001	177 (11.1)	490 (12.8)	< 0.1
Self-report very good and good general health	674 (41.3)	2789 (28.7)	< 0.001	652 (40.8)	1260 (33.0)	< 0.001
Number of comorbidities	1.7 ± 1.6	1.9 ± 1.7	< 0.001	1.7 ± 1.6	1.7 ± 1.6	0.81
Life satisfaction score	13.1 ± 3.5	12.7 ± 4.1	< 0.001	13.1 ± 3.5	12.8 ± 3.6	< 0.01
Completely independent in activities of daily Living	1620 (99.3)	9238 (95.2)	<0.001	1587 (99.2)	3722 (97.5)	<0.05
Sleep at night (hours)	6.4 ± 1.9	6.5 ± 1.9	0.86	6.4 ± 1.4	6.5 ± 1.7	0.43
Sleep at noon (minute)	40.0 ± 45.6	43.0 ± 50.1	< 0.05	39.6 ± 45.9	41.8 ± 48.0	0.12
Days of vigorous sport in a week	1.5 ± 2.6	1.8 ± 2.8	< 0.01	1.5 ± 2.6	1.9 ± 2.9	< 0.001
Days of moderate sport in a week	3.3 ± 3.1	2.7 ±3.2	< 0.001	3.4 ± 3.2	3.0 ± 3.2	< 0.001
Days of mild sport in a week	5.9 ± 2.2	5.3 ± 2.8	< 0.001	5.9 ± 2.2	5.6 ± 2.6	< 0.001
Attendance number of social activities	2.5 ± 1.5	0.8 ± 1.0	< 0.001	2.5 ± 1.5	0.9 ± 1.1	< 0.001

Values of continuous data are the mean ± SD, values of category data are n (%)

*PSM* propensity score matching, *CES-D* The Center for Epidemiologic Studies Depression Scale

Multilevel logistic regression was repeatedly conducted in the matching group by adjusting covariates step by step (Table 2). Model 1–2 showed that a significantly lower incidence of depression was related to WeChat usage without adjustment and adjustment for demographic information. The final model (model 3) showed that a significantly lower incidence of depression was related to WeChat usage after adjusting all the covariates (aOR: 0.76, 95% CI: 0.62–0.94). We also observed that a higher incidence of depression was significantly associated with individuals who were female, with a lower education level, lived in rural areas, had a lower income level, had worse general health, had lower life satisfaction, had disability, had more disease comorbidities, were less independent in daily activities, had less sleep at night, and had more vigorous activity.

**Table 2:** Multilevel regression results of effect of WeChat usage on depressive symptoms

	Model 1		Model 2		Model 3	
	OR (95%CI)	*P* value	aOR (95%CI)	*P*	aOR (95%CI)	*P* value
WeChat usage	0.65 (0.55 -0.77)	< 0.001	0.74 (0.63-0.89)	< 0.01	0.76 (0.62-0.94)	< 0.05
Age			1 (0.99-1.01)	0.94	0.99 (0.98-1)	< 0.1
Gender						
Male			Ref		Ref	
Female			1.3 (1.12-1.52)	<0.01	1.34 (1.08-1.66)	< 0.01
Race						
Non-minority			Ref		Ref	
Minority			1.29 (0.93-1.79)	0.13	1.21 (0.87-1.7)	0.26
Education						
Primary school			Ref		Ref	
Middle school			0.81 (0.7-0.95)	< 0.01	0.77 (0.65-0.9)	< 0.01
High school and above			0.68 (0.3-1.53)	0.353	0.67 (0.29-1.54)	0.35
Living area						
Urban			Ref		Ref	
Rural			1.42 (1.2-1.69)	< 0.001	1.5 (1.25-1.81)	< 0.001
Marital status						
Never married			Ref		Ref	
Married			0.22 (0.04-1.13)	<0.1	1 (0.17-5.77)	0.10
Divorced or separate			0.26 (0.05-1.41)	0.12	0.67 (0.11-3.95)	0.65
Income category						
≤25% (lower quartile)			Ref		Ref	
26%-50%			1.25 (0.87-1.82)	0.23	1.21 (0.82-1.8)	0.34
51%-75%			0.91 (0.59-1.43)	0.7	0.9 (0.56-1.44)	0.66
≥75%			0.7 (0.6-0.82)	< 0.001	0.73 (0.62-0.87)	< 0.001
Smoke						
Non-smoker					Ref	
Smoker					0.96 (0.79-1.16)	0.67
Drink						
Non-drinker					Ref	
Drinker					0.93 (0.84-1.03)	0.17
General Health					1.42 (1.29-1.57)	< 0.001
Life satisfaction					1.21 (1.18-1.25)	< 0.001
Disability					1.29 (1.05-1.6)	< 0.05
Comorbidity number					1.08 (1.02-1.13)	< 0.01
ADL					1.45 (1.14-1.85)	< 0.01
Sleep hour at night					0.88 (0.84-0.92)	< 0.001
Sleep minute at noon					1 (1.00-1.00)	0.79
Vigorous activity					1.03 (1.00-1.06)	< 0.05
Moderate activity					0.98 (0.96-1.01)	0.19
Mild activity					0.98 (0.95-1.01)	0.24
Social activities attendance					0.99 (0.93-1.06)	0.82
Constant	0.23 (0.21-0.26)	< 0.001	0.39 (0.06-2.4)	< 0.01	0.01 (0.0-0.09)	< 0.001

*aOR* adjusted Odds ratio, *ADL* Activities of Daily Living

**Fig. 2:**
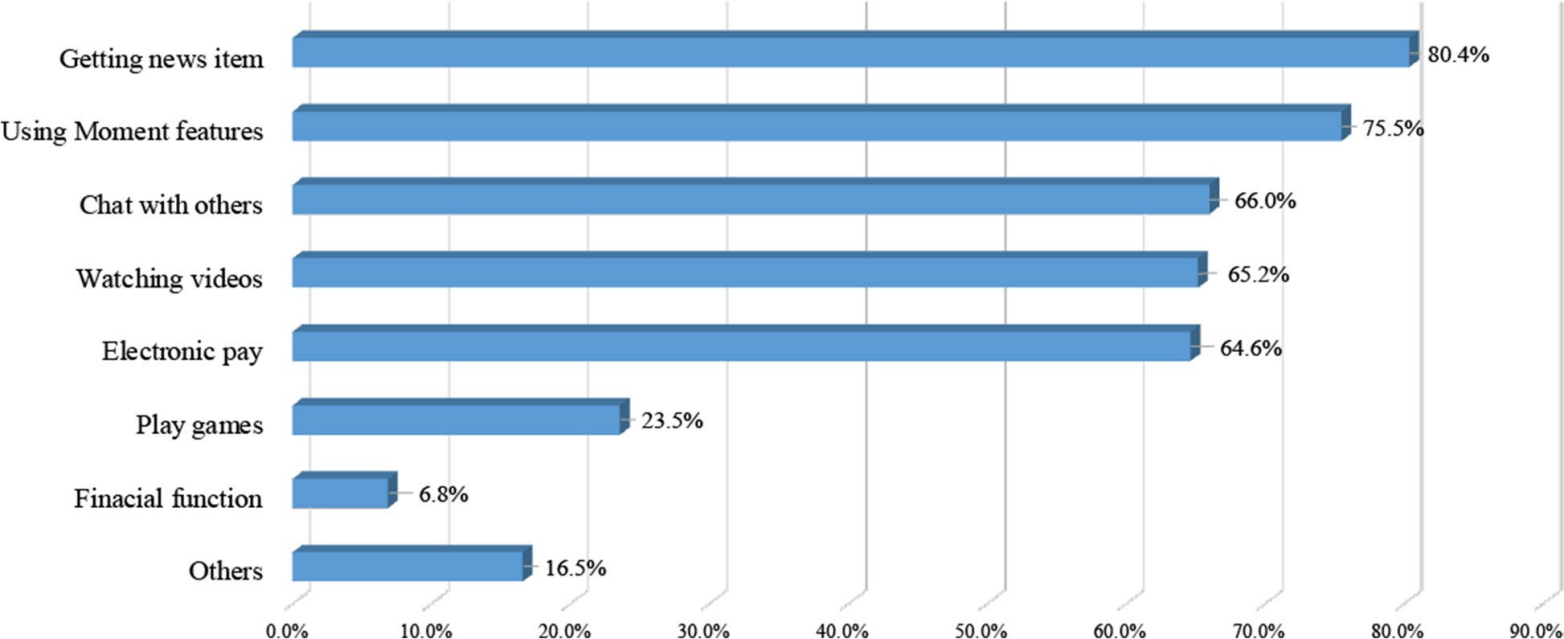
Functions used by WeChat users among Chinese adults aged ≥45 years old

Among the different WeChat user groups, only use WeChat was significantly associated with lower depression (aOR:0.68, 0.48–0.97) in the full model, but both WeChat and Moment users were not significantly associated with lower depression (aOR: 0.78, 0.60–1.01) at 0.05 level. See Supplementary Material S1 for full details. Among the WeChat users’ group (Fig. 2), the most common WeChat functions used were watching news (80.4%), followed by posting moment messages (75.5%) and chatting with friends (66.0%). Financial activities (6.8%) were the least commonly used feature. No association between the level and type of social media usage and depressive symptoms was observed in the full model. See Supplementary Material S2 for full details.

The sensitivity analysis yielded similar findings to the primary analyses. First, the logistic regression results indicated that WeChat usage was a protective factor against depression in the matching individuals (*N* = 5415, aOR:0.76, 95%CI: 0.62–0.94). See Supplementary Material S3 for full details. The results of multilevel logistic regression in the individuals aged > 60 years without dementia (*N* = 3467) were also similar to the results in the full model (aOR: 0.76, 95%CI: 0.59–0.97). See Supplementary Material S4 for full details. Our theoretical expectations were confirmed when this study changed the cutoff score for the determinant of depression from 10 to 9 (aOR: 0.71, 95%CI: 0.59–0.86) or 11 (aOR: 0.69, 95%CI: 0.55–0.86). See Supplementary Material S5 for full details.

## Discussion

The primary finding of this study identified that using social media WeChat was associated with a lower incidence of depressive symptoms among middle-aged and older populations using Chinese national survey data by multilevel logistic regression. We also found that the disparities in digital access existed among persons with different socioeconomic and health status. The study indicated that social media usage should not be neglected when making health policy to promote the mental health of the aging population in China.

This study found that social media usage had a solid positive effect on a lower incidence of depression among the target population after PSM and adjusted for common risk factors for depression, which was partially consistent with the previous studies. One of the recent reviews indicated that online social networking had advantages in decreasing depressive symptoms [45]. For the middle-aged and elderly population, contacting family and friends when they were geographically separated and entering intergenerational communication with younger family members are much more important needs [46]. Therefore, social media may improve information and emotional support among older people and is essential for reducing isolation and loneness [47]. Unlike the negative effect of social media usage among young populations [14, 18, 23], the effect among the old population may be more positive and milder. There may be two reasons to explain this difference. One is the purpose of social media use, and the other is the usage time. For the young population, seeking reassurance, social feedback, and technology-based social comparison was the primary purpose for social media use [48]. Higher self-esteem needs and fear of missing out made young people more vulnerable to stress associated with neglect and adverse reactions by online peers [49]. A higher risk of excessive social media usage time caused by lower self-control may lead to more emotional problems among young people [50]. However, more studies should conduct to test these assumed explanations about the difference of social media usage behavior between young and old population when data was available.

This study highlights that multipurpose social media can potentially improve psychological well-being in older adults. Interestingly, this finding indicated that a significantly lower incidence of depression was related to WeChat usage, despite type of individual functions used. It implied that a single function of social media might not work for improving mental health among the aging population. As a previous study suggested, touchscreen-based multipurpose apps, including health-, entertainment-, transportation-, and social media-related apps, may reduce the risk of depressive symptoms among older adults [51]. In addition, this finding suggested that those who were only using WeChat were significantly associated with lower depression, while WeChat and Moment users were not significantly associated with lower depression. The Moment usage seemed to eliminate the effect of WeChat usage on the lower rate of depression. Similar to the “Like”-function on Facebook or Instagram, WeChat allows users to comment and like posted moments of their friends via the Moment function. WeChat Moment uses intensity and received likes are positively associated with self-esteem [52]. Therefore, we supposed that the Moment users might have higher expectations of self-esteem and social comparison, which were reported as the leading causes related to depression through social media usage [53]. However, the data used in this study could not confirm the effect of the Moment function on depression. Further studies are needed using richer or qualitative data to explore “why” questions.

Social networks through internet users have increased rapidly in recent years in China. In 2011, only 6.5% of middle-aged and elderly individuals were reported as Internet users, and the rate increased to 20.4% in 2018 [54]. Although the rate increased rapidly when compared to the other developed countries, the social media use rate was still comparatively lower. For instance, the social media use rate was 64% among US adults aged 50–64 in 2018 [55]. The aging population is vulnerable to digital exclusion worldwide, especially during the COVID-19 outbreak [56]. Therefore, there are many challenges to making progress for eliminating the digital access gap in China. Our study was also consistent with previous studies in the socioeconomic disparities persistent with digital accessibility among middle-aged and older adults in China. A US study and German study indicated that persons with older age, living in rural areas, had lower education levels, and had lower income status had a significantly lower rate of using social media [57, 58]. A similar finding was identified in our study. An understanding of those differences would be greatly helpful to health policymakers in knowing what types of individuals they are likely to assist in digital time.

This finding implied the possible public health policy through eliminating digital disparities to make progress for mental disease prevention among the aging population in China. In the short run, increasing digital access through building basic telecommunication infrastructure in the lower developing areas was needed to eliminate the objective digital resource gap between urban and rural areas. On the individual level, guiding the middle-aged and older population, especially those who lived in the lower developing area, to properly use social media may have the potential to decrease their information loneness. Community support, digital training, and elders-friendly digital function development are also needed to increase healthy social media usage. In the long run, comprehensive methods to eliminate disparities in digital access, such as increasing primary education and generating income levels for lower socioeconomic populations in suburban and rural areas, may help maintain good mental health among the aging population [59].

This study has several limitations. First, we could not observe the effect of the intensity of WeChat usage on mental health status in this study sample because WeChat usage information was not collected in the initial survey of CHARLS data until 2018. Usage intensity, such as usage hours per day or per week, is considered as the second level of the digital divide which may impact mental health [60]. In addition, the effect of the purpose of WeChat usage was not fully analyzed in this study. The purpose may indicate user motivations and time spent on social media usage which is important for psychological status [61]. Although a positive effect of using WeChat is found in this study, according to previous studies, neither teenagers nor adults should be encouraged to expose themselves to the screen for too long. Future studies should focus on the effect of the social media usage time and purpose on the mental health of an aging population. Second, although we used statistical techniques to balance the individuals’ characteristics in the analysis, we could not establish the causal association between WeChat use and depression status. It was also difficult to identify the dynamic and long-run effect of WeChat usage on depression in this cross-sectional study. A longitudinal study, random clinical trials, or other causal inference should be encouraged to address the causal relationship between social media usage and mental health. Third, due to data restriction, we cannot estimate the effect of other social media, such as TikTok and WeiBo (a social media is like Twitter). The impact of other social media might be very different from that of WeChat. Future studies should explore the effect of other social media on mental health in a population. Fourth, although we adjusted as many covariates as possible in the analysis, some unobserved bias may exist. Future research is warranted to generalize our findings.

Despite these limitations, the present study has several strengths. First, we used national data, which made the results more generalizable among the middle-aged and elderly population in the whole country. To the best of our knowledge, this study was the first to address disparities of WeChat usage and examine the effects of WeChat on mental health among the middle-aged and elderly population in China. Second, compared to the previous studies, we used a double-adjustment method to control possible covariates, which made the estimations more accurate and robust.

## Conclusions

Our study suggested a significant association between using social media WeChat with lower incidence of depressive symptoms among people aged 45 and older in China. These findings have important implications for the possible prevention and intervention of depression using WeChat among the middle-aged and elderly population. Further studies need to be explored on the impact of other types of social media usage to improve mental health-related issues through social connections.

## Supplementary Information

**Additional file 1.**

## Data Availability

All data used in the study can be accessed through: https://charls.pku.edu.cn/en/ after registering by signing the agreement and provide real personal information for reviewing. No administrative permissions were required to access the raw data used in this study.

## Abbreviations

CES-D-10: 10-item form of the Center for Epidemiologic Studies Depression Scale
PSM: Propensity score matching
CHARLS: China Health and Retirement Longitudinal Study
COVID-19: Coronavirus Disease 2019
CSI-D: Community Screening Instrument for Dementia
ADL: Activities of Daily Living Scale
