# Supplementary material for: Association between using social media WeChat and depressive symptoms among middle-aged and older people: findings from a national survey
Source: BMC Geriatr. 2022 Apr 22;22:351. doi: 10.1186/s12877-022-03054-y (PMC9023108; doi:10.1186/s12877-022-03054-y)
Supplement: Supplementary file 1 — Additional file 1. [file 12877_2022_3054_MOESM1_ESM.docx]

Supplementary

Table S1 Multilevel regression results of association between WeChat user group and depression among population aged >45 years old

|  |  | OR (95% CI) | *P* |
| --- | --- | --- | --- |
| WeChat usage | |  |  |
|  | Group 1 | Ref |  |
|  | Group 2 | 0.68 (0.48-0.97) | <0.05 |
|  | Group 3 | 0.78 (0.6-1.01) | 0.063 |
| Age |  | 0.98 (0.96-0.99) | <0.01 |
| Gender |  |  |  |
|  | Male | Ref |  |
|  | Female | 1.22 (0.92-1.6) | 0.163 |
| Race |  |  |  |
|  | Non-minority |  |  |
|  | Minority | 1.22 (0.78-1.91) | 0.38 |
| Education | |  |  |
|  | Primary school | Ref |  |
|  | Middle school | 0.69 (0.56-0.86) | <0.01 |
|  | High school and above | 0.61 (0.26-1.4) | 0.242 |
| Living area | |  |  |
|  | Urban | Ref |  |
|  | Rural | 1.47 (1.17-1.85) | <0.01 |
| Marital status | |  |  |
|  | Never married | Ref |  |
|  | Married | 4.89 (0.39-61.07) | 0.218 |
|  | Divorced or separate | 2.95 (0.23-37.88) | 0.406 |
| Income category | |  |  |
|  | ≤25% (lower quartile) | Ref |  |
|  | 26%-50% | 1.05 (0.62-1.79) | 0.855 |
|  | 51%-75% | 0.71 (0.35-1.44) | 0.341 |
|  | ≥75% | 0.7 (0.56-0.87) | <0.01 |
| Smoke |  |  |  |
|  | Non-smoker | Ref |  |
|  | Smoker | 1.01 (0.79-1.29) | 0.933 |
| Drink |  |  |  |
|  | Non-drink | Ref |  |
|  | Drinker | 0.92 (0.81-1.05) | 0.234 |
| General Health | | 1.25 (1.1-1.42) | <0.01 |
| Life satisfaction | | 1.24 (1.19-1.29) | <0.001 |
| Disability |  | 1.54 (1.17-2.02) | <0.01 |
| Comorbidity number | | 1.1 (1.03-1.18) | <0.01 |
| Sleep hour at night | | 0.84 (0.78-0.89) | <0.001 |
| Sleep minute at noon | | 1 (1-1) | 0.27 |
| Vigorous activity | | 1.03 (1-1.07) | 0.08 |
| Moderate activity | | 0.98 (0.95-1.02) | 0.322 |
| Mild activity | | 0.96 (0.92-1) | 0.054 |
| Social activities attendance | | 1 (0.92-1.09) | 0.937 |
| ADL |  | 1.5 (1.1-2.04) | <0.01 |
| Constant |  | 0.01 (0-0.16) | <0.01 |

Note: We divided WeChat users into three groups: (1) Those with an answer of “no” to both questions assigned in group 1; (2) those with an answer of “yes” to WeChat usage but “no” to the Moment usage assigned in group 2, and (3) those with an answer of “yes” to both questions assigned in group 3.

Table S2 Multilevel regression results of association between WeChat user level and types and depression among population aged >45 years old

|  | | OR (95% CI) | P |
| --- | --- | --- | --- |
| WeChat usage level | | 0.93 (0.85-1.01) | 0.073 |
| WeChat use function type | |  |  |
|  | Social function | 1.18 (0.83-1.68) | 0.348 |
|  | Entertainment function | 0.99 (0.82-1.2) | 0.944 |
|  | Daily-life function | 0.85 (0.62-1.15) | 0.295 |
| WeChat use function | |  |  |
|  | Chat | 1.21 (0.84-1.74) | 0.313 |
|  | Watching the news | 0.77 (0.52-1.16) | 0.209 |
|  | Watching the video | 1.24 (0.87-1.77) | 0.239 |
|  | Play game | 0.92 (0.62-1.35) | 0.667 |
|  | Financial function | 1 (0.48-2.06) | 0.999 |

Note:

WeChat usage level: One WeChat function equaled to score 1, the total scores ranged from 0 to 6 with higher scores indicating higher levels of WeChat usage.

WeChat use function type: We divided Chat into social function; watching the news, watching the video, and playing games as entertainment function; financial and the others as the daily-life function.

WeChat use function: We put each WeChat function as a variable to see WeChat usage function exerts divergent effects on mental health.

Table S3 Logistic regression result of association between WeChat usage and depression in the matching group (N=5145)

|  |  | OR (95% CI) | *P* |
| --- | --- | --- | --- |
| WeChat usage | | 0.76 (0.62-0.94) | <0.05 |
| Age |  | 0.99 (0.98-1) | <0.05 |
| Gender |  |  |  |
|  | Male | Ref |  |
|  | Female | 1.35 (1.09-1.67) | <0.01 |
| Race |  |  |  |
|  | Non-minority | Ref |  |
|  | Minority | 1.22 (0.87-1.7) | 0.25 |
| Education | |  |  |
|  | Primary school | Ref |  |
|  | Middle school | 0.76 (0.64-0.89) | <0.01 |
|  | High school and above | 0.67 (0.29-1.54) | 0.35 |
| Living area | |  |  |
|  | Urban | Ref |  |
|  | Rural | 1.54 (1.28-1.85) | <0.001 |
| Marital status | |  |  |
|  | Never married | Ref |  |
|  | Married | 1.02 (0.18-5.91) | 0.98 |
|  | Divorced or separate | 0.67 (0.11-3.98) | 0.66 |
| Income category | |  |  |
|  | ≤25% (lower quartile of median) | Ref |  |
|  | 26%-50% | 1.2 (0.81-1.77) | 0.37 |
|  | 51%-75% | 0.88 (0.55-1.42) | 0.6 |
|  | ≥75% | 0.72 (0.61-0.85) | <0.001 |
| Smoke |  |  |  |
|  | Non-smoker | Ref |  |
|  | Smoker | 0.96 (0.8-1.16) | 0.7 |
| Drink |  |  |  |
|  | Non-drink | Ref |  |
|  | Drinker | 0.94 (0.85-1.04) |  |
| General Health | | 1.4 (1.27-1.55) | <0.001 |
| Life satisfaction | | 1.22 (1.18-1.26) | <0.001 |
| Disability |  | 1.28 (1.04-1.58) | <0.05 |
| Comorbidity number | | 1.09 (1.04-1.15) | <0.01 |
| ADL |  | 1.44 (1.14-1.81) | <0.01 |
| Sleep hour at night | | 0.88 (0.84-0.93) | <0.001 |
| Sleep minute at noon | | 1 (1-1) | 0.46 |
| Vigorous activity | | 1.03 (1-1.06) | <0.05 |
| Moderate activity | | 0.98 (0.96-1.01) | 0.17 |
| Mild activity | | 0.98 (0.95-1.01) | 0.19 |
| Social activities attendance | | 0.99 (0.92-1.06) | 0.72 |
| Constant |  | 0.01 (0-0.09) | <0.001 |

Table S4 Multilevel logistic regression result of association between WeChat usage and depression among population without dementia aged > 60 years (N=3467)

|  |  | OR (95% CI) | *P* |
| --- | --- | --- | --- |
| WeChat usage | | 0.76 (0.59-0.97) | <0.05 |
| Age |  | 1.02 (0.99-1.04) | 0.22 |
| Gender |  |  |  |
|  | Male | Ref |  |
|  | Female | 1.43 (1.1-1.86) | <0.01 |
| Race |  |  |  |
|  | Non-minority | Ref |  |
|  | Minority | 1.28 (0.86-1.9) |  |
| Education | |  |  |
|  | Primary school | Ref |  |
|  | Middle school | 0.73 (0.6-0.9) | <0.01 |
|  | High school and above | 1.01 (0.33-3.09) | 0.98 |
| Living area | |  |  |
|  | Urban | Ref |  |
|  | Rural | 1.66 (1.32-2.09) | <0.001 |
| Marital status | |  |  |
|  | Never married | Ref |  |
|  | Married | 2.79 (0.19-41.56) | 0.46 |
|  | Divorced or separate | 2.84 (0.18-43.67) | 0.46 |
| Income category | |  |  |
|  | ≤25% (lower quartile of median) | Ref |  |
|  | 26%-50% | 1.46 (0.89-2.38) | 0.13 |
|  | 51%-75% | 0.8 (0.44-1.47) | 0.48 |
|  | ≥75% | 0.79 (0.65-0.97) | <0.05 |
| Smoke |  |  |  |
|  | Non-smoker | Ref |  |
|  | Smoker | 0.96 (0.76-1.23) | 0.76 |
| Drink |  |  |  |
|  | Non-drink | Ref |  |
|  | Drinker | 1.00 (0.88-1.13) | 0.95 |
| General Health | | 1.37 (1.21-1.53) | <0.001 |
| Life satisfaction | | 1.2 (1.15-1.25) | <0.001 |
| Disability |  | 1.3 (1-1.68) | <0.05 |
| Comorbidity number | | 1.07 (1.01-1.15) | <0.05 |
| ADL |  | 2.81 (1.51-5.19) | <0.01 |
| Sleep hour at night | | 0.86 (0.81-0.92) | <0.001 |
| Sleep minute at noon | | 1 (1-1) | 0.72 |
| Vigorous activity | | 1.01 (0.98-1.04) | 0.53 |
| Moderate activity | | 0.99 (0.96-1.02) | 0.71 |
| Mild activity | | 1 (0.96-1.04) | 0.92 |
| Social activities attendance | | 1.03 (0.95-1.12) | 0.44 |
| Constant |  | 0 (0-0.02) | <0.001 |

Table S5 Multilevel logistic regression result of association between WeChat usage and depression in the matching group when CES-D score >9 and >11(N=5145)

|  | | **CES-D score >9** | | **CES-D score >11** | |
| --- | --- | --- | --- | --- | --- |
|  | | OR (95% CI) | *P* | OR (95% CI) | *P* |
| WeChat usage | | 0.71 (0.59-0.86) | <0.01 | 0.69 (0.55-0.86) | <0.01 |
| Age | | 1.00 (0.99-1.01) | 0.43 | 1.00(0.99-1.02) | 0.5 |
| Gender | |  |  |  |  |
|  | Male | Ref |  |  |  |
|  | Female | 1.31 (1.08-1.59) | <0.01 | 1.52 (1.21-1.9) | <0.001 |
| Race | |  |  |  |  |
|  | Non-minority | Ref |  |  |  |
|  | Minority | 1.21 (0.89-1.65) | 0.23 | 1.23 (0.86-1.76) | 0.26 |
| Education | |  |  |  |  |
|  | Primary school | Ref |  |  |  |
|  | Middle school | 0.72 (0.62-0.84) | <0.001 | 0.71 (0.6-0.84) | <0.001 |
|  | High school and above | 0.57 (0.27-1.21) | 0.15 | 0.61 (0.25-1.5) | 0.28 |
| Living area | |  |  |  |  |
|  | Urban | Ref |  |  |  |
|  | Rural | 1.45 (1.22-1.71) | <0.001 | 1.66 (1.36-2.02) | <0.001 |
| Marital status | |  |  |  |  |
|  | Never married | Ref |  |  |  |
|  | Married | 1.59 (0.28-9.02) | 0.60 | 0.92 (0.16-5.22) | 0.92 |
|  | Divorced or separate | 1.09 (0.19-6.28) | 0.93 | 0.6 (0.1-3.48) | 0.57 |
| Income category | |  |  |  |  |
|  | ≤25% (lower quartile) | 0 (0-0) |  |  |  |
|  | 26%-50% | 0.98 (0.67-1.43) | 0.92 | 0.87 (0.56-1.35) | 0.53 |
|  | 51%-75% | 0.91 (0.59-1.4) | 0.66 | 0.82 (0.5-1.34) | 0.43 |
|  | ≥75% |  | <0.001 | 0.7 (0.59-0.84) | <0.001 |
| Smoke | |  |  |  |  |
|  | Non-smoker | Ref |  |  |  |
|  | Smoker | 0.95 (0.8-1.14) | 0.60 | 0.97 (0.79-1.19) | 0.78 |
| Drink | |  |  |  |  |
|  | Non-drink | Ref |  |  |  |
|  | Drinker | 0.98 (0.9-1.08) | 0.69 | 0.99 (0.89-1.1) | 0.83 |
| General Health | | 1.38 (1.26-1.51) | <0.001 | 1.36 (1.23-1.51) | <0.001 |
| Life satisfaction | | 1.21 (1.18-1.25) | <0.001 | 1.24 (1.2-1.28) | <0.001 |
| Disability | | 1.2 (0.99-1.46) | <0.01 | 1.31 (1.06-1.63) | <0.05 |
| Comorbidity number | | 1.09 (1.04-1.15) | <0.001 | 1.09 (1.03-1.15) | <0.01 |
| ADL | | 1.42 (1.13-1.79) | <0.01 | 1.51 (1.2-1.91) | <0.01 |
| Sleep hour at night | | 0.87 (0.83-0.91) | <0.001 | 0.9 (0.86-0.95) | <0.001 |
| Sleep minute at noon | | 1 (1-1) | 0.94 | 1 (1-1) | 0.88 |
| Vigorous activity | | 1.03 (1.01-1.06) | <0.05 | 1.05 (1.02-1.08) | <0.01 |
| Moderate activity | | 0.98 (0.96-1.01) | 0.14 | 0.97 (0.94-0.99) | <0.05 |
| Mild activity | | 0.98 (0.95-1.01) | 0.12 | 0.98 (0.95-1.01) | 0.26 |
| Social activities attendance | | 0.96 (0.9-1.02) | 0.19 | 0.95 (0.88-1.03) | 0.2 |
| Constant | | 0.01 (0-0.07) | <0.001 | 0 (0-0.02) | <0.001 |
